# Correction: Watrin-Avino et al. Affect Recognition, Theory of Mind, and Empathy in Preschool Children with Externalizing Behavior Problems—A Group Comparison and Developmental Psychological Consideration. *Children* 2023, *10*, 1455

**DOI:** 10.3390/children12040405

**Published:** 2025-03-24

**Authors:** Laura M. Watrin-Avino, Franziska J. Forbes, Martin C. Buchwald, Katja Dittrich, Christoph U. Correll, Felix Bermpohl, Katja Bödeker

**Affiliations:** 1 Department of Child and Adolescent Psychiatry, Charité Campus Virchow, Charité—Universitätsmedizin Berlin, 13353 Berlin, Germany; f.forbes@outlook.de (F.J.F.); martin.buchwald@charite.de (M.C.B.); katja.dittrich@outlook.com (K.D.); christoph.correll@charite.de (C.U.C.); katja.boedeker@charite.de (K.B.); 2 The Zucker Hillside Hospital, Department of Psychiatry, Northwell Health, Glen Oaks, NY 11004, USA; 3 Donald and Barbara Zucker School of Medicine at Hofstra/Northwell, Department of Psychiatry and Molecular Medicine, Hofstra University, Hempstead, NY 11549, USA; 4 The Feinstein Institutes for Medical Research, Center for Psychiatric Neuroscience, Northwell Health, New Hyde Park, NY 11030, USA; 5 DZPG, German Center for Mental Health, Partner Site Berlin, 10785 Berlin, Germany; 6 Department of Psychiatry and Neuroscience, Charité Campus Mitte, Charité—University Medicine Berlin, 10117 Berlin, Germany; felix.bermpohl@charite.de

## 1. Errors in Tables and Text

In the original publication [1], there was a mistake in the composition of the sample. Subsequently, one participant had to be excluded from the sample because she did not fulfil the inclusion criteria of the CBCL. The corrected results for *N* = 49 are presented in Tables 1–3 and in the following data-related text passages:

Abstract;
2.3.2. Psychiatric Diagnoses;
3.1. Study Sample;
3.2. Descriptive Statistics;
3.3. Intercorrelation of Variables Examined, Pooled across Study Groups;
5. Strengths and Limitations.

see the specific modifications below.

## 2. Corrected Tables

Table 1, Table 2 and Table 3 are corrected for *N* = 49. The correct captions, content and legends appear below.

We have inserted the correct Legend A for Table 1 and the Bonferroni-corrected significance level in Legend C for Table 3—each below the tables.

**Table 1:** Demographic and clinical characteristics of the samples.

Sample Characteristics	*n*	Clinical Sample *M* (*SD*)	*n*	Control Sample *M* (*SD*)	*df*	χ^2^ or *t*	*p*-Value
Sex							
Male	19		14		1, 47	6.566	0.0104
Female	3		13				
Age (month)	22	70.14 (11.21)	27	63.96 (8.80)	47	2.161	0.0359
Socioeconomic status (SES)	19	12.46 (3.16)	26	14.74 (1.63)	24.99	2.878	0.0081
CBCL externalizing problems	22	71.36 (7.66)	27	47.37 (6.68)	47	11.716	<0.0001
CBCL internalizing problems	22	60.50 (7.99)	27	46.89 (10.69)	47	4.946	<0.0001
WPPSI-III matrix reasoning (gen. IQ)	22	9.91 (3.25)	27	10.85 (2.43)	38.09	1.128	0.2664
WPPSI-III language	22	19.59 (3.96)	27	22.52 (2.90)	47	2.985	0.0045
WPPSI-III active vocabulary	22	9.36 (2.46)	27	11.52 (1.60)	47	3.694	0.0006
WPPSI-III passive vocabulary	22	10.23 (1.90)	27	11.00 (1.82)	47	1.450	0.1538

Legend A. CBCL: Child Behavior Checklist; gen. IQ: general intelligence quotient; WPPSI: Wechsler Preschool and Primary Intelligence Scale. Means (*M*) are presented with standard deviations (*SD*) in parentheses. χ^2^ for analysis of sex. *t* for all other analyses. WPPSI-III scale value scores: *M* = 10, *SD* = 3; WPPSI-III language scale value score: *M* = 20, *SD* = 6.

**Table 2:** Intercorrelation of social cognition and behavioral variables, pooled across groups.

Social Cognition and Behavioral Variables	1	2	3	4	5	6	7	8
1 Affect recognition	1	0.048	0.008	0.103	0.121	−0.094	−0.203	−0.333 *
2 Emotion comprehension (affective theory of mind)		1	0.535 **	0.013	0.070	0.119	−0.181	−0.010
3 Cognitive theory of mind			1	0.103	0.301 *	0.476 **	−0.441 **	−0.192
4 Empathy Emotion contagion				1	0.434 **	0.365 **	−0.108	0.105
5 Empathy Attention to others’ feelings					1	0.488 **	−0.316 *	−0.253
6 Empathy Proscocial action						1	−0.490 **	−0.257
7 Externalizing problems							1	0.630 **
8 Internalizing problems								1

Legend B. * *p* < 0.05; ** *p* < 0.01.

**Table 3:** ANCOVAs with age, sex, and language as covariates.

Domains of Social Cognition	Clinical Sample (*n* = 22) *M* (*SD*)	Control Sample (*n* = 27) *M* (*SD*)	*df*	F	*p*-Value *	Part. η*^2^*	Power 1 − ß
Cognitive theory of mind	3.41 (1.33)	4.74 (1.02)	1, 47	16.161	0.0002	0.269	0.97
Affective theory of mind	5.05 (1.81)	5.74 (1.77)	1, 47	1.849	0.1808	0.040	0.09
Emotion contagion	0.37 (0.30)	0.45 (0.26)	1, 47	0.322	0.5733	0.007	0.05
Attention to others’ feelings	1.31 (0.47)	1.62 (0.31)	1, 47	13.622	0.0006	0.236	0.51
Affect recognition	8.86 (2.80)	10.56 (2.49)	1, 47	1.335	0.2542	0.029	0.31
Prosocial action	0.81 (0.50)	1.21 (0.37)	1, 47	13.309	0.0007	0.232	0.67

Legend C. Means (*M*) are presented with standard deviations (*SD*) in parentheses. * Bonferroni adjusted significance level: *p* = 0.05/6 = 0.008. Part. η^2^ = 0.01 small effect, η^2^ = 0.06 moderate effect, η^2^ = 0.14 strong effect.

## 3. Corrected Text

As a result, the following data-related text passages had to be amended.

- Abstract

Sample description modified for *N* = 49 follows.

The text “Compared to 28 HCs, 22 preschoolers with EBPs (total sample mean_age_ = 5.5 years +/− 0.8 years, range= 4.2–6.9 years, males 66%) had significantly greater impairments in cognitive ToM (*p* = 0.0012, η^2^ = 0.266), attention to others’ feelings (*p* = 0.0049, η^2^ = 0.222), and prosocial action (*p* = 0.0070, η^2^ = 0.210), each representing strong effect sizes. EBPs were significantly related to cognitive domains, like prosocial action (*r* = −0.501), cognitive ToM (*r* = −0.425), and attention to others’ feelings (*r* = −0.332), but not to affective domains of social cognition” has been corrected to “Compared to 27 HCs, 22 preschoolers with EBPs (total sample mean_age_ = 5.6 years +/− 0.8 years, range= 4.2–6.9 years, males 67%) had significantly greater impairments in cognitive ToM (*p* = 0.0002, η^2^ = 0.269), attention to others’ feelings (*p* = 0.0006, η^2^ = 0.236), and prosocial action (*p* = 0.0007, η^2^ = 0.232), each representing strong effect sizes. EBPs were significantly related to cognitive domains, like prosocial action (*r* = −0.490), cognitive ToM (*r* = −0.441), and attention to others’ feelings (*r* = −0.316), but not to affective domains of social cognition”.

- 2.3.2. Psychiatric Diagnoses

The listing of diagnoses was corrected in this section. The correct text appears below.

The text “five children” has been corrected to “four children” (two places).

- 3.1. Study Sample

The modified sample composition for *N* = 49 follows.

The text “6 children were excluded from the analyses (one clinical subject and five controls)” has been corrected to “7 children were excluded from the analyses (one clinical subject and six controls)”.

The text “28 control subjects” has been corrected to “27 control subjects”.

- 3.2. Descriptive Statistics

The corrected descriptions of the samples for *N* = 49 are shown below.

In the first paragraph, the text “(*t*(48) = 10.500, *p* < 0.001). The same was true for internalizing problem scores (*t*(48) = 4.338, *p* < 0.001)” has been corrected to “(*t*(47) = 11.716, *p* < 0.0001). The same was true for internalizing problem scores (*t*(47) = 4.946, *p* < 0.0001)”.

In the second paragraph, the text “(χ^2^(1) = 7.260, *p* = 0.007, φ = 0.381), age (*t*(48) = 2.338, *p* = 0.024), SES (*t*(24.85) = 2.739, *p* = 0.010), and active vocabulary (*t*(48) = 3.551, *p* < 0.001). In receptive vocabulary (*t*(48) = 1.595, *p* = 0.117) and general IQ (*t*(37.69) = 1.228, *p* = 0.227)” has been corrected to “(χ^2^(1) = 6.566, *p* = 0.0104, φ = 0.366), age (*t*(47) = 2.161, *p* = 0.0359), SES (*t*(24.99) = 2.878, *p* = 0.0081), and active vocabulary (*t*(47) = 3.694, *p* < 0.0006). In receptive vocabulary (*t*(47) = 1.450, *p* = 0.1538) and general IQ (*t*(38.09) = 1.128, *p* = 0.2664)”

- 3.3. Intercorrelation of Variables Examined, Pooled across Study Groups

Modified correlations between EBP and the social-cognitive domains for *N* = 49 follow.

In the first paragraph, the text “low correlations were found for the cognitive domains, such as cognitive ToM (*r* = −0.425) and empathy-related attention to others’ feelings (*r* = −0.332), and moderate correlations were found for prosocial action (r = −0.501)” has been corrected to “moderate correlations were found for the cognitive domains, such as empathy-related attention to others’ feelings (*r* = −0.316) and cognitive ToM (*r* = −0.441), as well as for prosocial action (*r* = −0.490)”

The modified results of the ANCOVAs for *N* = 49 follow here.

In the second list item, the text “(*F*(1,49) = 16.34, *p* = 0.0012, η^2^ = 0.266)” has been corrected to “(*F*(1,47) = 16.161, *p* = 0.0002, η^2^ = 0.269)”.

In the third list item, the text “(*F*(1,49) = 12.83, *p* = 0.0049, η^2^ = 0.222) and prosocial action (*F*(1,49) = 12.00, *p* = 0.0070, η^2^ = 0.210)” has been corrected to “(*F*(1,47) = 13.622, *p* = 0.0006, η^2^ = 0.236) and prosocial action (*F*(1,47) = 13.309, *p* = 0.0007, η^2^ = 0.232)”.

The modified power of the ANCOVAs for *N* = 49 follow.

In the third paragraph, the text “As shown in Table 3, with a relatively small sample size, a power [50] well above 0.8 could be achieved for three variables with large effect sizes (prosocial action: 0.95, cognitive ToM: 0.99, attention to others’ feelings: 0.96). Unfortunately, this cannot be said for the variables with small effects (affect recognition, affective ToM) or without appreciable effects (emotion comprehension), which would have required a larger sample size” has been corrected to “As shown in Table 3, with a relatively small sample size, a power [50] well above 0.8 could be achieved for cognitive theory of mind (power 1 − ß = 0.97). However, the power of two empathy variables fell below the desired level of 1 − ß = 0.8 (attention to others’ feelings: 0.51, prosocial action: 0.67). Nevertheless, these variables still show high significance. For the other variables with small effects (affective theory mind, affect recognition) or no appreciable effects (emotion contagion), a larger sample size would have been required”.

- 5. Strengths and Limitations

The modified effect sizes of the ANCOVAs for *N* = 49 follow.

In the second paragraph, the text “0.95–0.99” has been corrected to “η^2^ = 0.232–0.269”.

The authors state that the scientific conclusions are unaffected. This correction was approved by the Academic Editor. The original publication has also been updated.
